# Factors influencing medical students’ choice of specialization: A gender based systematic review

**DOI:** 10.1016/j.eclinm.2020.100589

**Published:** 2020-10-24

**Authors:** Mathieu Levaillant, Lucie Levaillant, Nicolas Lerolle, Benoît Vallet, Jean-François Hamel-Broza

**Affiliations:** a Methodologic and biostatistics department, CHU Angers, University Angers, France; b Department of Pediatric Endocrinology and Diabetology, University Hospital of Angers, Angers Cedex 9, France; c Medical Intensive Care Unit, CHU Angers, Angers University Hospital, Angers, France; d Univ. Lille, CHU Lille, ULR 2694 - METRICS: Évaluation des technologies de santé et des pratiques médicales, F-59000 Lille, France

**Keywords:** Gender study, Medical specialties, Public policy

## Abstract

**Background:**

Students’ choice of medical specialties has evolved throughout year, with a growing interest in quality of life and in technological specialties. We investigated the repartition of such choices in the world and its influencing factors with a focus on the gender's influence, for helping policy-makers to deal with medical shortage and territorial to specialty disconnect.

**Methods:**

A systematic search was conducted on MEDLINE and Scopus from January 2010 to January 2020. Data extraction and analysis followed JBI and PRISMA recommendations. The selected articles had to focus on medical students, detail their choice of specialty, and look for factors influencing their choice. Articles were excluded if they only assessed the attractiveness of a specialty, or evaluated a public policy. This review was registered on PROSPERO, CRD 42020169227.

**Findings:**

751 studies were screened, and fifty-four were included. Surgery and internal medicine were the most wanted specialties, both in occidental and non-occidental countries. The main factors influencing the choice of specialty were lifestyle, work-life balance and discipline interest, with variation across different countries. Gender clearly affected this choice with 63.7% of men willing radiology and 14.7% of men in obstetrics and gynecology.

**Interpretation:**

Influential factors vary with specialty and are affected by the country of residence. Gender has a great impact in students’ willingness to work in specific specialties. Policymakers should adapt their appealing strategies according to the country and the medical discipline concerned.

**Funding:**

The authors have no support or funding to report.

**Research in context**

**Evidence before this study**

All around the world, new medical knowledge may induce a subspecialisation and a disconnect between specialists and population needs, probably aggravated by changes in the men-to-female ratio of caregivers.

**Added value of this study**

Factors influencing choice of medical specialty may change either from a specialty or a country to another, and appear quite different in occidental and non-occidental countries. Gender lead to different expectations about working conditions, therefore variations in specialty choices.

**Implications of all the available evidence**

Policymakers need to adapt their attracting strategies according to the specificity of the willingness of students in their country and the medical discipline concerned.

Alt-text: Unlabelled box

## 1 Introduction

Even if the number of physicians is higher than ever, the subspecialisations induced by new medical knowledge leads to a disconnect between specialists working in the world and population needs [1]. Many students entering school with a career plan [2], [3], [4] often evolved throughout the course of their studies [4].

Medical students’ choices have evolved throughout time, with a growing interest in quality of life or in technological excellence [5]. Not only thoughtful individual factors such as interest for the discipline, work-life balance or role modeling can influence the specialization choice, but also sociobiological aspects. For example, gender can modify factors associated with the choice of several medical specialties: in the US, men-to-female ratio was 4.9:1 in obstetrics and gynecology [6], 24.6% of last-year male medical students would chose internal medicine compared with 11.8% female in Rwanda [7], or in Korea, where 2.9% of male would be interested in paediatrics for 10.7% of woman [8]. Men are more interested by technical challenges, salary, career and prestige, women by time related aspects and societal orientation [9], [10], [11].

A shift in the gender ratio in medical students have occurred at the end of the 20th century [12] from a male to a female over-representation, leading to difficulties for policymakers to adapt incentives, learning programs, schemes for managing physicians’ flows and the specialization's issue. If many papers have investigated the gender imbalance among physicians [6], [7], [8], only a few have looked for a gender-impact on students willingness before their specialty choice. This gender-ratio shift could lead to a change in the repartition of health workers and increase the disconnection between population's need and adequate care.

The purpose of this study was to investigate the factors influencing specialty’ choice of medical students in the world, with emphasis on gender and differences between occidental and non-occidental countries.

## 2 Methods

A systematic review following PRISMA guidelines was undertaken to explore the factors influencing the choice of specialty among medical students and the influence of the gender. The review was registered on PROSPERO, no. 42020169227.

### 2.1 Search strategy

We developed a search strategy according to Joanna Briggs Institute (JBI) reviewers manual [13] based on recommendations for systematic review of etiology and risk. Search was made on the 3 may of 2020, in MEDLINE and Scopus, from the 1st of January 2010 to the 1st of January 2020. Only the last decade was used to include articles, as the gender distribution could change over time [14].

MEDLINE and Scopus databases were screened using the keywords “speciality”, “career choice”, “motivation”, “interest”, “ambition”, “influence”, “factor” and “medical students” (*search strategy in supplementary* Table 1).

### 2.2 Screening of literature

Screening and data extraction were done independently by two researchers (ML and JFH). In case of disagreement, a third researcher was involved (LL). The literature was firstly screened by title and abstract according to the inclusion and exclusion criteria. The remaining full-text articles were assessed for final inclusion.

### 2.3 Inclusion and exclusion criteria

For being included, the study had to focus on medical students after their selection year and before their specialization, detail the choice of specialty of each student, and look for factors influencing their choice.

Articles only referring to students before being admitted to medical studies, exploring students perceptions of specialties independently of their willingness, assessing practices’ manner interest, evaluating a public policy such as incentives to choose a specialty or not being an English original study (i.e., protocol publication, letters, or comments) were excluded. No methodological criteria were applied for articles selection, Qualitative and quantitative paper could be collected.

If requested data was not available or further details were needed, the original study's authors were reached by email.

Data was extracted following an *a priori* defined grid (*supplementary file,* Table 2). Extracted information included author, year of publication, research design and objectives, information about the reviewed study, and the specific information contributing to this review's central question. Extraction form was tested on five studies by each reviewer to ensure that all relevant results were extracted.

The quality of the included studies was assessed through Ariëns et al.’s score [15] modified for reviews focusing on influencing factors [16] (*supplementary file,* Table 3). This score is suitable either for qualitative and quantitative studies.

### 2.4 Classification of specialities and factors influencing their choice

Fifteen different categories of specialties were *a priori* defined: anaesthesiology and intensive care, dermatology, ear, nose and throat (ENT), emergency room, general practice (GP), internal medicine, obstetrics and gynecology (O&G), oncology, ophthalmology, paediatrics, pathology, psychiatry, radiology, social medicine & public health and surgery.

Seven pre-defined groups of influencing factors, based on a literature review of the topic, were explored along with gender: lifestyle and work-life balance (factors about doctors’ schedule and the balance between happiness in a job and in every day's life), societal orientation (willingness to have an important work for the community and the population in needs), prestige and income (wage and place of a specialty in the community or among colleagues), place of practice (place where practicing, possibility to have a career in public or private facility), scope of practice (diversity of a specialty, possibility to perform a wide panel of acts), role model and university influence (students’ academic background and influence of teachers on his choices) and interest toward the discipline (*supplementary file*, Table 4).

A factor was considered as influencing a specialty choice either in a positive or a negative way.

### 2.5 Analysis procedure

Data analysis was planned to be stratified according to gender and origin country, dichotomized by occidental (OC) and non-occidental countries (NOC) according to the distinction made by S. Huntington [17]. OC’ category relate to North America, European Union, Australia and New-Zealand. NOC have been treated as a global category because of the foreseeable under-representation of these countries in our study [12].

For the gender analysis, we aggregated data from the papers in which full numbers of men and women interested in a particular discipline were provided for calculating the proportion of men as the number of men interested in a specialty divided by all the people interested in it.

Finally, we performed a post-hoc analysis by country's income level according to World Bank ranking [18], either comparing low income with high income countries, and by stratifying OC and NOC by income level.

### 2.6 Role of the funding source

This study did not benefit from any funding.

## 3 Results

**Fig. 1:**
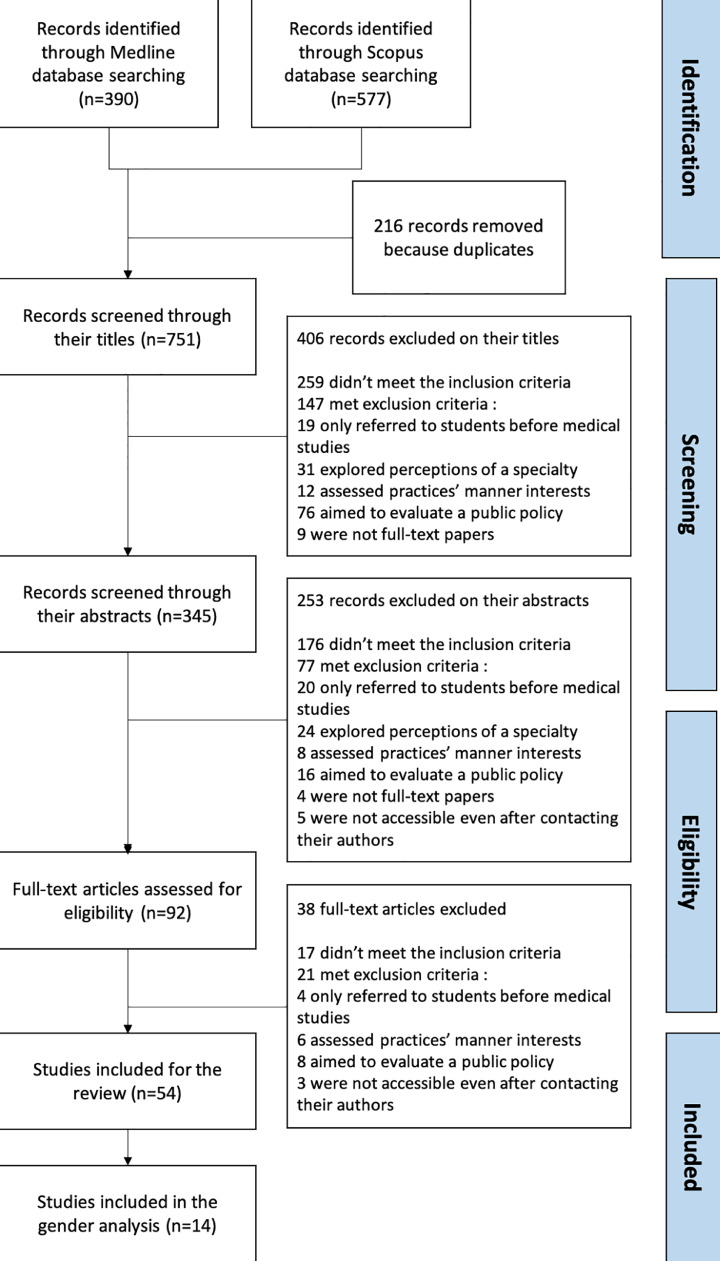
Flowchart.

577 articles were identified through the Scopus database, and 390 through the MEDLINE database. 751 articles remained after removing the duplicates. 406 articles were excluded based on their title, 253 based on their abstract and 38 based on the full-text (Fig. 1).

The 54 articles included in the systematic review were all cross-sectional and observational: 50 were quantitative studies, three were qualitative and one was both (Table 1). 23 studies were performed in OC (42.6%) and 31 in NOC (57.4%), for a total of 29 different countries involved. Most of studies (92.6%) were based on a survey addressed toward medical students in different countries. The mean response rate (RR) of all surveys included in this review was 69.1% (61.0% in OC and 73.7% in NOC), with a total sample size of 26 270 students surveyed. The mean age of the respondents was 23.9 years (25.6 in OC, 23.0 in NOC). The gender percentage was 48.5% men (42.8% in OC, 52.7% in NOC).

**Table 1:** Included studies assessing the factors influencing the choice of specialty among medical students around the world.

First author and year	Country	Design	Sample size	Period of study	Response rate	Men percentage	Mean age	Methodology assessment	Quality
**Abdulrahman M. 2016**[19]	United Arab Emirates (NO)	QT	956	All undergraduate students	46.0%	44.6%	NA	14	Good
**Akhund S. 2012**[20]	Pakistan (NO)	QT	148	Semester 1, 3, 7, 9	43.7%	54.0%	20.5 (2.2)	11	Average
**Al-Fouzan R. 2012**[21]	Kuwait (NO)	QT	387	All except first year	91.7%	42.9%	21.45 (1.72)	17	Very good
**Al-Mendalawi MD. 2010**[22]	Iraq (NO)	QT	108	Final year	91.5%	64.8%	NA	14	Good
**Alahwal H.M.S. 2010**[23]	Saudi Arabia (NO)	QT	151	Interns	60.0%	66.0%	NA	12	Average
**Alawad AA. 2015**[24]	Sudan (NO)	QT	647	First to fifth year	73.0%	38.6%	NA	11	Average
**Alenezi M. 2019**[25]	Saudi Arabia (NO)	QT	75	Interns	NA	52.0%	24.49 (2.15)	11	Average
**Alkhaneen H. 2018**[26]	Saudi Arabia (NO)	QT	436	Second and third phases	53.4%	57.0%	NA	12	Average
**AlKhilaiwi RM. 2018**[27]	Saudi Arabia (NO)	QT	236	Fifth and final year	78.7%	64.8%	NA	11	Average
**Alshahrani M. 2014**[28]	Saudi Arabia (NO)	QT	379	Fourth, fifth and sixth year	58.0%	33.3%	NA	12	Average
**Alsubaie N. 2016**[29]	Saudi Arabia (NO)	QT	252	Second and third year	81.8%	50.4%	NA	14	Good
**Anand R. 2019**[30]	India (NO)	QT	364	Bachelor	79.1%	37.9%	NA	13	Good
**Anna Muscatello MR. 2017**[4], [5], [6], [7][31]	USA (O)	QT	335	Forth year	93.0%	52.9%	24.3 (2.1)	12	Average
**Azizzadeh A. 2003**[32]	USA (O)	QT	111	Forth year	69.0%	60.8%	NA	14	Good
**Barber S. 2018**[33]	UK (O)	QT & QL	280	Final and penultimate years	89.0%	51.0%	NA	14	Good
**Bien A. 2019**[34]	Germany (O)	QT	361	Fourth, fifth or final year	70.9%	33.4%	NA	12	Average
**Bilal M. 2018**[35]	Pakistan (NO)	QT	1400	Final year	100%	33.0%	24	14	Good
**Chen YC. 2014**[36]	Taiwan (NO)	QT	405	Interns	91.4%	59.4%	26.81 (3.44)	17	Very good
**Correia Lima de Souza L. 2015**[37]	Brazil (NO)	QT	1225	Medical students and doctors	79.2%	37.5%	24	14	Good
**Diderichsen S. 2013**[38]	Sweden (O)	QT	372	Final year	89.0%	42.0%	27.5	15	Good
**Du J. 2009**[39]	New Zealand (O)	QT	87	First to fifth year	0.25%	37.0%	NA	11	Average
**Enoch L. 2013**[40]	USA (O)	QT	145	Forth year	88.0%	54.0%	26.2 (1.6)	12	Average
**Fehlmann A. 2019**[41]	Switzerland (O)	QT	1749	Final year	56.0%	37.0%	NA	15	Good
**Grasreiner D. 2018**[42]	Germany (O)	QT	720	First to sixth year	13.1%	24.9%	24	11	Average
**Gutierrez-Cirlos C. 2019**[43]	Mexico (NO)	QT	697	Final year	81.0%	35.0%	24 (1)	13	Good
**Hamid S. 2019**[44]	Pakistan (NO)	QT	314	Fourth and final year	54.5%	NA	22.63 (1.473)	12	Average
**Ibrahim M. 2014**[45]	UK (O)	QT	641	Final year and graduate	12.0%	44.0%	NA	12	Average
**Ie K. 2018**[46]	Japan (NO)	QT	1408	Final year students'	74.0%	66.3%	24	14	Good
**Kawamoto R. 2016**[47]	Japan (NO)	QT	368	First to fifth year	88.2%	61.7%	21.4 (3.6)	14	Good
**Kazzi AA. 2001**[48]	USA (O)	QT	393	NA	76.0%	NA	28 (3)	12	Average
**Khader Y. 2008**	Jordan (NO)	QT	440	Second fourth and sixth year	77.7%	64.0%	21.1 (2.0)	14	Good
**Khater-Menassa B. 2005**[49]	Lebanon (NO)	QT	127	Final year	97.0%	74.0%	25	15	Good
**Kiolbassa K. 2011**[50]	Germany (O)	QT	1299	All years	11.0%	40.0%	24.1 (3.1)	12	Average
**Kumar R. 2011**[51]	India (NO)	QT	282	All years	74.4%	89.0%	20.89	13	Good
**Kuzman M.R. 2014**[52]	Croatia (O)	QT	122	Final year	61.0%	36.0%	24.38 (0.819)	13	Good
**Lam CY. 2016**[53]	Hong-Kong (NO)	QT	233	All medical graduates	73.7%	47.6%	23	16	Very good
**Lefevre JH. 2010**[11]	France (O)	QT	1780	Sixth year	68.8%	38.0%	23.8 (1.4)	16	Very good
**Lefèvre JH. 2010**[54]	France (O)	QT	1742	Sixth year	67.0%	38.0%	23.8 (1.4)	16	Very good
**Lydon S. 2015**[55]	Ireland (O)	QT	334	Medical students and doctors	NA	50.7%	NA	12	Average
**Mehmood SI. 2013**[56]	Saudi Arabia (NO)	QT	590	First to fifth year	92.5%	57.0%	21.5 (2.5)	14	Good
**Newton DA. 2005**[57]	USA (O)	QT	1334	Fourth year	73.0%	51.0%	28.1 (3.2)	11	Average
**Onyemaechi N. 2017**[58]	Nigeria (NO)	QT	152	Final year	98.0%	72.4%	25.8 (2.5)	14	Good
**Osborn HA. 2007**[59]	Canada (O)	QT	323	Fourth year	59.0%	46.7%	26	12	Average
**Pianosi K. 2016**[60]	Canada (O)	QL	70	NA	NA	NA	NA	9	Poor
**Querido S. 2018**[61]	Netherlands (O)	QL	24	Final year	NA	16.7%	NA	10	Poor
**Rouhani M. 2017**[62]	UK (O)	QT	137	All years	NA	38.0%	NA	11	Average
**Saigal P. 2007**[63]	Japan (NO)	QL	NA	NA	NA	NA	NA	9	Poor
**Scott AJ. 2017**[64]	South Africa (NO)	QT	245	First to sixth year	24.4%	44.0%	21.4	12	Average
**Ster MP. 2017**[65]	Slovenia (O)	QT	343	Final year	NA	38.5%	24.9	16	Very good
**Subba S.H. 2012**[66]	India (NO)	QT	373	Fourth, sixth and eight semester	NA	50.4%	20.2 (1.6)	9	Poor
**Sutton PA. 2014**[67]	UK (O)	QT	482	Final year	NA	41.0%	NA	13	Good
**Wang K.-I. 2007**[68]	Taiwan (NO)	QT	185	Fifth to seventh year	92.5%	NA	NA	14	Good
**Wu S.M. 2014**[69]	Hong-Kong (NO)	QT	247	Fifth year	93.9%	54.5%	23 (1.49)	17	Very good
**Zarkovic A. 2006**[70]	New Zealand (O)	QT	256	Final year - One to Fourth year postgraduate	64.0%	51.4%	NA	12	Average

O: Occidental country, NO: Non-occidental country.

USA: United States of America, UK: United Kingdom, NA: Not Available.

All studies were cross-sectional and observational study. QT: Quantitative, QL: qualitative.

When available, ages are expressed in years, with mean and, when provided, standard deviation into brackets.

Quality grade are assessed through the Ariens et al.’s score, and range from 0 (worst methodological quality) to 17 (best one).

Seven studies (3 OC and 4 NOC) were rated of very good quality with a methodological score of 16 to 17 points. 21 studies scored between 14 and 15 and ranked good quality. 22 studies scored between 11 and 13 points and ranked average quality and 4 had a poor assessment.

### 3.1 Influencing factors

Factors influencing students’ choices were studied depending on each discipline. The most frequent factor was lifestyle and work-life balance, quoted by 33 studies (60.0%) as an important factor. Interest in the discipline and gender were quoted respectively by 25 (45.5%) and 21 (38.2%) studies. Other factors appeared in less than a third of the studies.

**Fig. 2:**
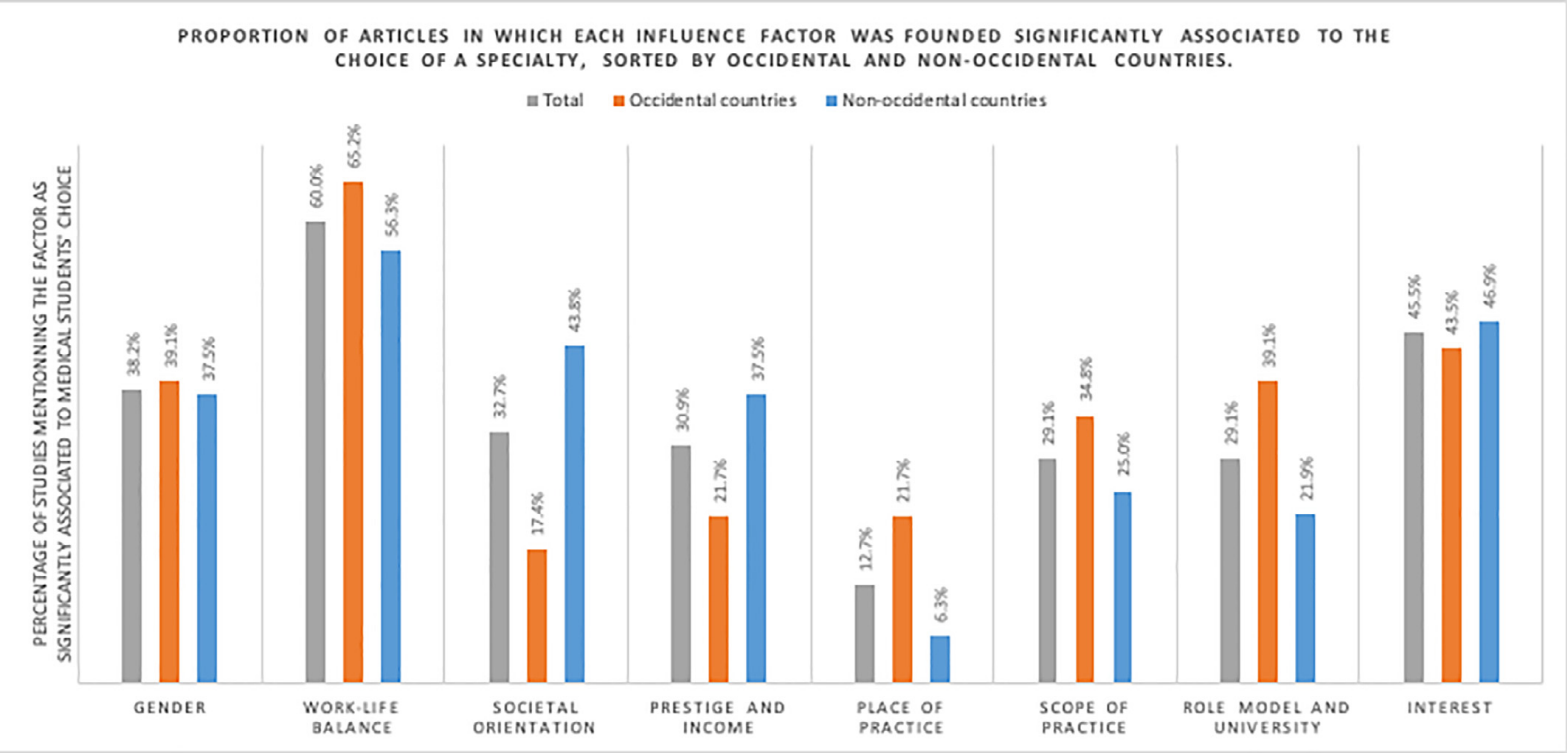
Proportion of articles in which each influence factor was founded significantly associated to the choice of a specialty, sorted by occidental and non-occidental countries. Each percentage is referring to the number of the article quoting the studied factor as influencing the students’ choice, either in occidental or non-occidental countries.

Societal orientation among with prestige and income was more important in NOC studies (respectively 43.8% VS 17.4% and 37.5% VS 21.7%) whereas place of practice and role modeling or academic status were pointed out principally in OC studies (respectively 21.7% VS 6.3% and 39.1% VS 21.9%) (Fig. 2).

Surgery was the most attractive specialty according to medical students followed by Internal Medicine, either in OC and NOC.

Attractiveness of some specialties varied depending on origin country: GP attended the third place in OC (10.5%) and the sixth in NOC (5.3%). Dermatology and Social Medicine were more attractive in NOC (4.0% VS 0.4% and 1.4% VS 0.2% respectively), and anaesthesiology in OC (6.3% VS 3.8%).

The influence of work-life balance and interest for the discipline differed greatly depending on the country. Work-life balance had a bigger impact on the choice of surgery, general practice, psychiatry, anaesthesiology, emergency room and social medicine mostly in OC, and on pathology mostly in NOC. The interest for the discipline influenced the choice of internal medicine, psychiatrists, anaesthesiology only in OC.

Gender had an impact on the choice of surgery, O&G and GP mostly in OC, and on O&G and paediatrics in NOC (*supplementary*Table 5).

As the country income level could be an important confounder in our analyses, we stratified them depending on the country income level, according to World Bank ranking [18]. 23 OC and 23 NOC were high-income. No changes were observed for the three first influencing factors either for OC or NOC, and the first three specialties chosen were also the same. The percentage of men did not show any major difference. The main differences were for the second and the third most wanted specialty. According to NOC, the top three were Surgery (64.1%), Emergency Room (62.9%) and Radiology (62.5%), whereas for high-income NOC, they were Surgery (57.5%), ENT (55.6%) and Anesthesiology (48.8%). No additional analyses were performed in the specific low-income level countries, as the low-income level countries were systematically NOC.

**Fig. 3:**
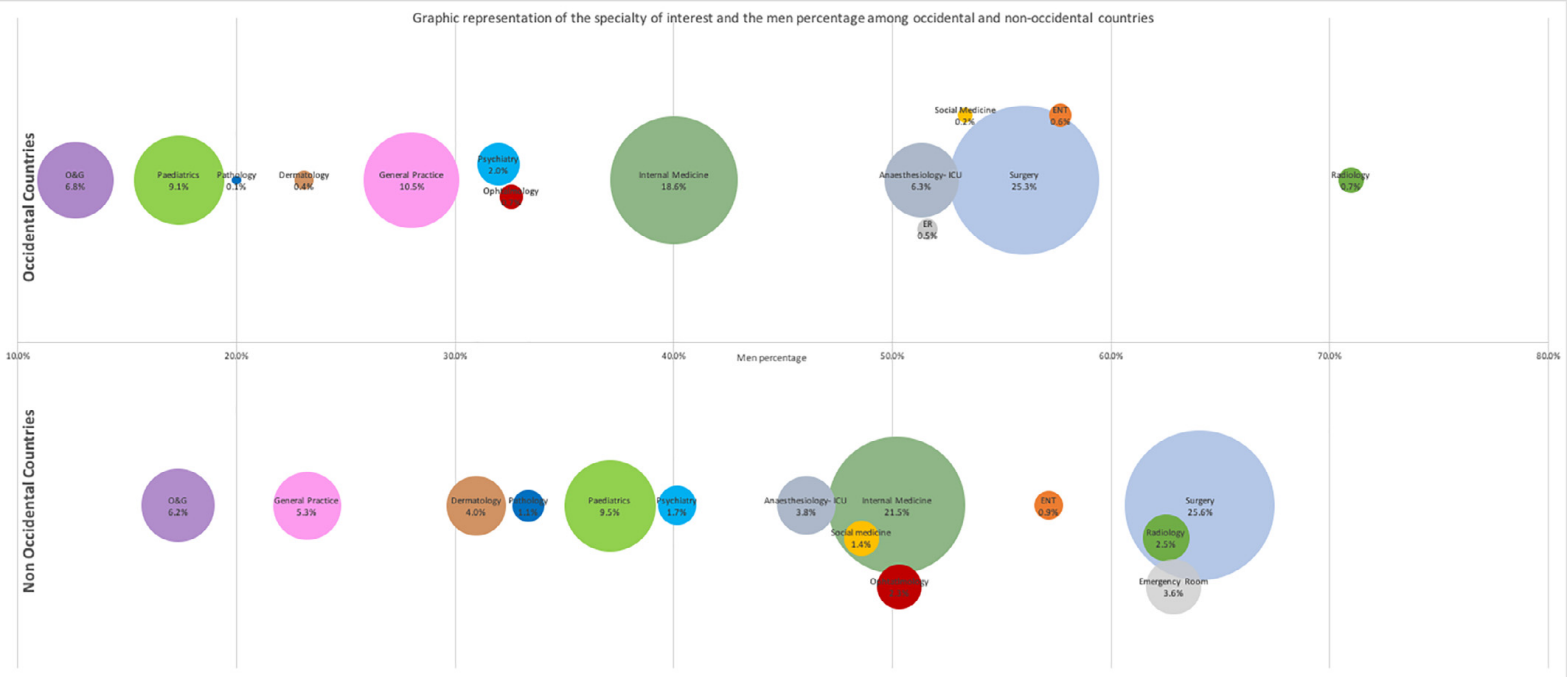
Graphic representation of the specialty of interest and the men percentage among all countries. Above the abscissa axis are represented the men percentage and proportion of medical students’ interests among occidental countries. Below the axis, the same data are provided for non-occidental countries. Abscissa axis represents the percentage of men among each specialty. The size of each bubble reflects the relative proportion of medical students’ interest in a specialty. The bubbles have been vertically distributed to allow a better readability, without another meaning of the ordinate axis. For example, radiology interested 0.7% of medical students’, and mostly men (above70% men percentage) in occidental countries, and 2.5% in non-occidental countries, with less men interested (between 60% and 65% men percentage). *ER: Emergency Room, ICU: Intensive Care Unit, O&G:* Obstetrics and Gynaecology*.*

Fourteen of the included studies reported data concerning the association between the interest in a specialty and the gender, 5 from OC and 9 from NOC. Specialties attracting a higher proportion of men were radiology (men percentage (MP)=63.7%), surgery (MP=60.9%), emergency medicine (MP=57.6%) and ENT (MP=57.3%), whereas those interesting mostly women were O&G (MP=14.7%), GP (MP=26.9%), (paediatrics MP=27.3%) and dermatology (MP=29.6%) (Fig. 3).

The highest gaps between NOC and OC MP among countries were found for radiology, anaesthesiology, ophthalmology and paediatrics. Radiology and anaesthesiology had higher MP in OC (71% and 51.3%) than in NOC (62.5% and 46.1%) whereas MP were higher in NOC for paediatrics (37.1% in OC and 17.4% in NOC) and ophthalmology (50.3% in NOC and 32.6% in OC).

Men were mainly interested in surgery and internal medicine, both in OC and NOC, with a higher preference for surgery in NOC (45.8% VS 25.3%).

**Fig. 4:**
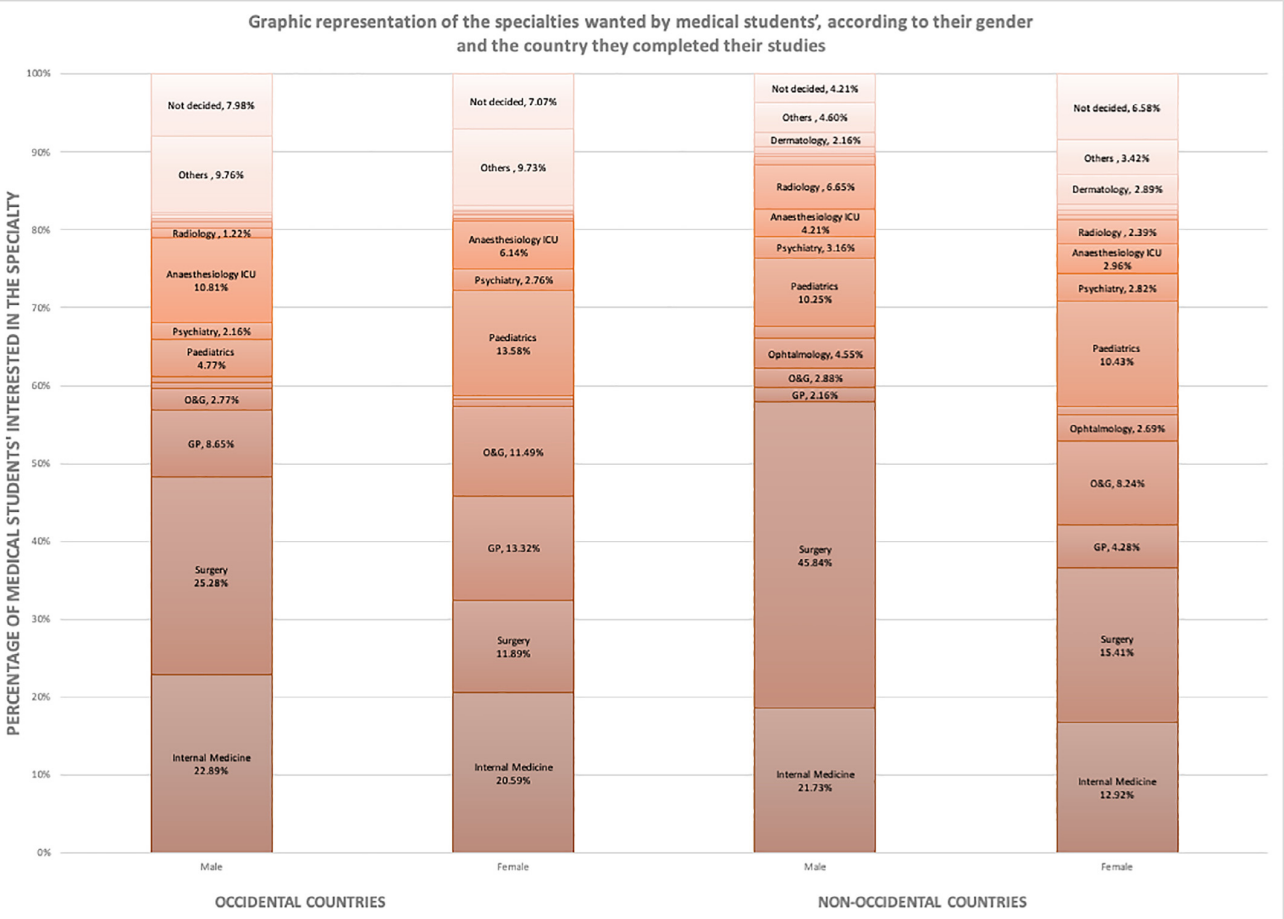
**Graphic representation of the specialties wanted by medical students’, according to their gender and the country they completed their studies**. *Percentages lower than 1% are not reported on the graph*. *ICU: Intensive Care Unit, O&G: Obstetrics and Gynaecology, ENT: Ear, Nose, Throat*.

Both in OC and in NOC, women were more attracted by O&G (respectively 11.5% VS 2.8% and 8.2% VS 2.9%). In OC, women were much more interested in paediatrics than men (13.6% vs 4.8%). Such a difference was not highlighted in NOC (10.3% vs 10.4%). Finally, GP was found more appealing in OC than in NOC, both for men and women (respectively 8.6% and 13.3% in OC and 2.2% and 4.3% in NOC) (Fig. 4).

## 4 Discussion

In this review including 23 occidental and 31 non-occidental studies, lifestyle and work-life balance such as interest for the discipline happened to be frequently quoted as affecting the selection of a specialty. Gender was also a key factor with a high percentage of women in O&G (MP=14.7%) and a high percentage of men in surgery (MP=61.0%).

Influencing factors were difficult to isolate from career choice. Career decision-making is an evolving process. Querido et al. showed that first-year students were more personally oriented (geography, self-confidence, positive attitude toward patient population) compared to final-year students interests (lifestyle, workload, personal experiences) [16].

Other studies have shown a raise of lifestyle factors for students’ specialty choice [71]. This growing of lifestyle importance must be taken into account by policymakers because the less attractive a specialty is, the more important the shortage will be, and so the workload. The frequent appearance of interest as an influencing factor, more than prestige or income argues against a possible benefit from only raising physician's salary to deal with the challenge of access to care.

Gender influence - an important aim of our review - has rarely been investigated in previous research [12]. We showed that women ratio was higher in specialties whose choices are affected by interest for the discipline and societal orientation, while the men ratio was higher in technical disciplines, mostly linked to lifestyle and income, confirming the results of studies showing that career decision-making process is influenced by gender [4,10,11,44,72]. Although women seem to prefer primary care careers over medical specialty, linked with better work-life balance and wider practice's types, this is no longer only a women's issue, the family argument becoming just as important for men [38,73].

Gender ratio might be interpreted with caution, as for country whose population gender ratio might not be 1:1, having such a medical sex ratio around 1:1 may illustrate an over-representation of a specific gender. For example, the proportion of men in Kuwait medical students could be estimated at 42.9% [18], while the proportion of men in the whole Kuwait population was 58.1% [74].

In our review, OC and NOC countries representation was fairly balanced. Societal orientation along with prestige and income appeared mainly in NOC studies whereas place of practice and role modeling or academic status were pointed out principally in OC studies. Men were more interested in radiology and anaesthesiology in OC than in NOC, and more interested in ophthalmology and paediatrics in NOC than in OC.

Many confounders may exist when analyzing the impact of OC and NOC countries, especially since our categorization gather in a same group China, Japan, the India subcontinent and Arabia. However, taking into account the income-level of the countries (one of the most important confounders) did not change the estimated impact of the type of country. Even though, these results should not be extrapolated to each different NOC, as political or cultural factors that may be essential in the specialty choice process have not been investigated. Even if socio-demographic factors other than gender may also affect specialty choice [75], such factors like ethnicity need to be contextualized: a specific ethnicity can be predominant in a country and stand for a minority in another, and so were not included in this systematic review analysis.

One strength of our overview is not only presenting factors with a known association with medical career decision-making in general but also with a specialty-specific career preference. These results may be taken into account by public authorities to adapt their access to care policies: GP may not be attracted by higher wages nor academic career, whereas surgeons might be. Longitudinal study should be realized for assessing the evolution of factors influencing students until the definitive choice.

This study should be considered with limitations. First, specialties and influencing factors categories can suffer from lack of precision. Another categorization could have provided slightly different results. Specialties categorization chosen may bias gender's estimation as they aggregate subspecialties with different gender repartition. For example, surgery stands either for orthopaedics (1 men for 5.9 women in 2016 in the US) and general surgery (1 men for 1.7 women in 2016 in the US), with very different gender percentage [6]. The studied influential factors may not be exhaustive. Relevant information can be missing, as the willingness of train or practice abroad . Specialty choice may be driven by the possibility for students to practice abroad, or by the needs of the countries they want to work in.

Secondly, students considered in our review were at different stages of their training. Perception may change during training, young students having more interest for income and prestige than young doctors [76]. Analyzing by stages of medical education or running a prospective follow-up study could help in avoiding this bias, but was not possible in this study.

Thirdly, our work did not consider the specific medical demographic condition of each country, specialty may be more attractive for students’ according to the number of already existing practitioners. Students’ willingness may also be driven by the global health context, guessing that after the COVID-19 outbreak, specialists in infectious disease and intensivists will be more attractive.

Moreover, our study does not separate public and private medical schools, neither the length of training for each subspecialty, especially students’ debt could impact their choice for shorter-trained subspecialties.

Comparing data between specialties and countries might have been limited because each country may not offer the same type of subspecialties neither the same status with it. For example, wages can be 2 to 10 times higher from a country to another, and could change influencing factors [77]. Also, a GP or a surgeon might not have the same work organization in every country.

For all studies included in the data synthesis, the levels of methodological quality was assessed by Soethout et al. checklist [16]. These criteria however slightly favor quantitative studies over qualitative ones. Qualitative studies can only obtain a maximum score of 15 where quantitative studies can obtain a maximum of 17 points. The results from studies with a low methodical quality are properly expected more uncertain and should be interpreted carefully. Even though, when excluding all studies with a methodological score under 13, results were slightly the same and ranks of factors influencing medical students’ choice did not vary.

To conclude, influencing factors change either from a specialty or a country to another. Factors that influence a medical students’ choice of specialty are not the same in occidental countries than in others. Gender have an important impact in students’ choice of specialty. Policymakers need to adapt their attracting strategies according to the specificity of the willingness of students in their country and the medical discipline concerned. Further investigations looking on the imbalance between future needs and specialists repartition could also be helpful.

## Declaration of Competing Interest

We declare no competing interest

## Appendix Supplementary materials

Image, application 1

Image, application 2
