# Supplementary material for: Factors influencing medical students’ choice of specialization: A gender based systematic review
Source: eClinicalMedicine. 2020 Oct 24;28:100589. doi: 10.1016/j.eclinm.2020.100589 (PMC7588859; doi:10.1016/j.eclinm.2020.100589)
Supplement: Supplementary file 2 [file mmc2.docx]

**Factors influencing medical students’ choice of specialization: a systematic gender based review**

**Supplementary files**

| **Supplemental table 1 – Keywords and query** | |
| --- | --- |
| Database | **Keywords and query** |
| Medline | Keywords: speciality, career choice, motivation, interest, ambition, influence, factor, medical, student  Query: (((specialization [Title/Abstract]) OR (specialty [Title/Abstract]) OR (speciality [Title/Abstract])) AND (("career choice" [Title/Abstract]) OR (motivation* [Title/Abstract]) OR (interest* [Title/Abstract]) OR (ambition* [Title/Abstract]) NOT (selection [Title/Abstract])) AND ((influence* [Title/Abstract]) OR (factor* [Title/Abstract])) AND ((medical [Title/Abstract]) AND (student* [Title/Abstract])) AND English[lang]) |
| Scopus | Keywords: speciality, career choice, motivation, interest, ambition, influence, factor, medical, student  Query: (TITLE-ABS-KEY ((specialization OR specialty OR speciality) AND ("career choice" OR motivation* OR interest* OR ambition*) AND (influence* OR factor*) AND "medical student*")) AND NOT (selection*) AND (LIMIT-TO (LANGUAGE, "English ")) |

| Supplemental table 2 – Extraction data form | |
| --- | --- |
| Title | ___________________________________ |
| Author | ___________________________________ |
| Year of publication | ___________________________________ |
| Research design | ___________________________________ |
| Research objectives | ___________________________________ |
| 1. Characteristics of the population |  |
| Country | ___________________________________ |
| Continent (occident versus non occidental country) |  Occident  Non-occident |
| Year of the study | ___________________________________ |
| Sample size | ___________________________________ |
| Response rate | ___________________________________ |
| Gender ratio | ___________________________________ |
| Student’s grade | ___________________________________ |
| 2. Number of students interested in each of the 15 pre-defined specialty (sorted by sex) | ___________________________________ |
| 3. Factors influencing the choice of specialty | ___________________________________ |
| Three major factors influencing (based on the  7 predefined) the choice of specialty | ___________________________________ |
| Gender influence |  Yes  No |
| 4. Other information | ___________________________________ |
|  |  |

**Supplemental table 3: Methodology assessment of the included articles, based on Ariëns et al’s method.**

1. Study purpose

- 2 points if a specific purpose was clearly described

1. Study design

- 2 points if the study design was clearly described

1. Response rate

- 3 points if > 80% or between 65 and 80% with a non-response survey carried out
- 2 points if RR between 65 and 80% or between 50 and 65% with a non-response survey carried out
- 1 point if RR between 50 and 65%

1. Kind of survey

- 3 points if national survey or > 80% of the country’s centers
- 2 points if regional survey or > 50% of the country’s centers
- 1 point if local survey or > 10% of the country’s centers

1. Number of investigated factors

- 3 points if more than 2 factors associated with the choice of medical career were investigated
- 2 points if 2 factors associated with the choice of medical career were investigated
- 1 point if 1 factor

1. Career or profession

- 2 points if actual medical career of profession was investigated

1. Statistical analysis

- 2 points if a statistical analysis was performed for the outcome

The content is a seven category list of criteria: (a) study purpose, (b) study design, (c) response rate, (d) nature of the survey, (e) number of investigated factors (associated factors and career factors), (f) career or professional, and (g) statistical analysis. Four items (a, b, f, and g) could be awarded 0 or 2 points and three items (c, d, and e) could be awarded 0–3 points, depending on the match between content and the criteria. It ranges from 0 (worst methodological quality) to 17 (best one).

# Supplemental table 4: Categories of factors influencing the choice of specialty among medical students in each study

| **Lifestyle and work-life balance** | Flexibility outside of medicine |
| --- | --- |
|  | Acceptable hours of practice |
|  | Flexibility inside of medicine |
|  | Acceptable on-call schedule |
|  | Keeping options open |
| **Societal orientation** | Health promotion |
|  | Long term relationship with patients |
|  | Focus on patients in a community |
|  | Social commitment |
| **Prestige and income** | High income potential |
|  | Adequate income to eliminate debt |
|  | Status among colleagues |
|  | Stable and secure future |
|  | Financial reward |
| **Place of practice** | Focus on urgent care |
|  | Focus on in-hospital care |
|  | Private practice |
| **Scope of practice** | Results of interventions immediately available |
|  | Wide variety of patient problems |
|  | Subspecialties available |
| **Role model and university influence** | Meaningful past experience with a physician |
|  | Emulate a physician |
|  | Adequate competencies |
|  | Perceived academic level |
| **Interest** | Interest in the specialty |
|  | Good match to the career |

|  | **Type of country** | **Number of studies concerned** | **Gender** | **Lifestyle and work-life balance** | **Societal orientation** | **Prestige and ambition** | **Place of practice** | **Scope of practice** | **Role model and university influence** | **Interest** |
| --- | --- | --- | --- | --- | --- | --- | --- | --- | --- | --- |
| **Internal medicine** | OC | **3** |  | **✔** | **✔** |  |  |  |  | **✔** |
|  | NOC | 3 | **✔** |  |  |  |  | **✔** | **✔** |  |
| **Surgery** | OC | **10** | **✔** | **✔** | **✔** | **✔** | **✔** |  | **✔** | **✔** |
|  | NOC | 6 | **✔** | **✔** |  | **✔** |  | **✔** | **✔** | **✔** |
| **Ears, Nose, Throat (ENT)** | OC | **1** |  |  |  |  |  |  |  |  |
|  | NOC | 1 |  |  |  |  |  |  |  |  |
| **General practice** | OC | **7** | **✔** | **✔** | **✔** |  | **✔** | **✔** | **✔** | **✔** |
|  | NOC | 6 |  | **✔** | **✔** |  | **✔** | **✔** | **✔** | **✔** |
| **Obstetrics and Gynecology** | OC | **4** | **✔** |  |  |  | **✔** | **✔** | **✔** | **✔** |
|  | NOC | 4 | **✔** | **✔** |  |  |  | **✔** | **✔** | **✔** |
| **Paediatrics** | OC | **3** |  | **✔** |  |  | **✔** | **✔** |  | **✔** |
|  | NOC | 4 | **✔** | **✔** | **✔** | **✔** |  | **✔** | **✔** | **✔** |
| **Psychiatry** | OC | **3** |  | **✔** | **✔** |  |  |  | **✔** | **✔** |
|  | NOC | 1 |  |  |  |  |  |  |  |  |
| **Anaesthesiology & Intensive care** | OC | **1** |  | **✔** |  |  |  |  |  | **✔** |
|  | NOC | 2 |  | **✔** |  | **✔** |  | **✔** |  |  |
| **Emergency medicine** | OC | **1** |  | **✔** |  |  | **✔** | **✔** |  |  |
|  | NOC | 2 |  |  |  | **✔** |  |  |  |  |
| **Pathology** | OC | **0** |  |  |  |  |  |  |  |  |
|  | NOC | 1 |  | **✔** |  |  |  |  |  |  |
| **Dermatology** | OC | **0** |  |  |  |  |  |  |  |  |
|  | NOC | 2 |  |  |  |  |  |  |  |  |
| **Social medicine** | OC | **1** |  | **✔** |  |  |  |  |  | **✔** |
|  | NOC | 2 | **✔** |  |  |  |  |  |  | **✔** |

**Supplemental Table 5: Factors influencing the choice of specialty according to the country of study.**

Ophthalmology, Radiology and Oncology not presented because of the absence of concerned studies
